# Expectations of nursing personnel and physicians on dementia training: A descriptive survey in general hospitals in Germany and Greece

**DOI:** 10.1007/s00391-019-01625-0

**Published:** 2019-10-15

**Authors:** Julia Schneider, Mara Gkioka, Sotirios Papagiannopoulos, Despina Moraitou, Brigitte Metz, Magdalini Tsolaki, Andreas Kruse, Birgit Teichmann

**Affiliations:** 1 Network Aging Research, Heidelberg University, Bergheimer Straße 20, 69115 Heidelberg, Germany; 2 School of Medicine, Aristotle University of Thessaloniki, University Campus, 54124 Thessaloniki, Greece; 3 3rd Department of Neurology, Aristotle University of Thessaloniki, Thessaloniki, Greece; 4 Lab of Psychology, Section of Experimental & Cognitive Psychology, School of Psychology, Aristotle University of Thessaloniki, Thessaloniki, Greece; 5 Klinik für Geriatrie und Geriatrisches Zentrum Karlsruhe, Diakonissenkrankenhaus Karlsruhe-Rüppurr, ViDia Christliche Kliniken Karlsruhe, Karlsruhe, Germany; 6 1st Department of Neurology, AHEPA University Hospital, Thessaloniki, Greece; 7 Insitute of Gerontology, Heidelberg University, Heidelberg, Germany

**Keywords:** Dementia, Education, Hospitals, Healthcare personnel, Needs, Demenz, Krankenhaus, Schulung, Personal, Bedarf

## Abstract

**Background:**

The number of dementia training programs in hospital settings is steadily increasing. The way training sessions are designed influences the way the learning content is implemented in practice. To develop a successful training it is important to meet the needs of the target group; however, not much is known about staff preferences and expectations relevant to future dementia training programs in hospitals in Germany and Greece.

**Objective:**

The aim of this survey was to explore staff training needs relevant to the topic of dementia, in general hospitals in Germany and Greece. This study analyzed the interests of staff members, preferences and expectations with respect to dementia training.

**Material and methods:**

This was a descriptive survey based on a 54-item questionnaire conducted with 61 nursing staff, head nurses and physicians (Germany: *n* = 25, Greece: *n* = 36) recruited from 5 hospitals (Germany: *n* = 3, Greece: *n* = 2). Parts of the questionnaire explored participants’ previous education regarding dementia and their expectations towards future dementia programs.

**Results:**

Although staff attendance in educative programs was high in the last 5 years for both countries, participation in dementia training programs was low (Germany 24%, Greece 5.5%). Additionally, the great majority of participants were willing to be trained in future dementia training programs (Germany 96%, Greece 100%). Employees from both countries expect increased clinical skills as a result of participation in such training programs. In Greece, staff members hope for better handling of people with dementia, while in Germany, concrete practical advice is preferred.

**Conclusion:**

There seems to be a strong willingness to participate in further dementia training programs where not only theoretical knowledge is provided but also practical advice.

**Electronic supplementary material:**

The online version of this article (10.1007/s00391-019-01625-0) contains supplementary material, which is available to authorized users.

## Abstract (de)

**Hintergrund:**

Die Zahl der Demenzschulungen in Krankenhäusern steigt stetig an. Wie die Schulungen durchgeführt werden, beeinflusst die Umsetzung des Gelernten in der Praxis. Bei der Schulungsentwicklung sollten Bedürfnisse und Erwartungen der Zielgruppe beachtet werden. Über die Erwartungen von Klinikpersonal aus Deutschland und Griechenland an zukünftige Demenzschulungen ist wenig bekannt.

**Ziel:**

Ziel der Studie ist es, den Schulungsbedarf von Klinikpersonal zum Thema Demenz in Deutschland und Griechenland zu untersuchen. Analysiert werden Interessen, Präferenzen und Erwartungen der Mitarbeiter an die Teilnahme an einer Demenzschulung.

**Material und Methoden:**

Es handelt sich um eine deskriptive Studie, die auf einem 54-Item-Fragebogen basiert. Es nahmen 61 Pflegekräfte, Stations‑/Bereichsleitungen und Ärzte (Deutschland, DE: *n* = 25, Griechenland, GR: *n* = 36) aus 5 Krankenhäusern (DE: *n* = 3, GR: *n* = 2) an der Studie teil. Ein Teil des Fragebogens untersuchte die bisherige Teilnahme und Erwartungen an eine Demenzschulung.

**Ergebnisse:**

In beiden Ländern war die allgemeine Teilnahme an Schulungen in den letzten 5 Jahren hoch, jedoch besuchten nur wenige eine Schulung zum Thema Demenz (DE: 24 %, GR: 5,5 %). Beinahe alle Probanden waren bereit, an einer zukünftigen Demenzschulung teilzunehmen (DE: 96 %, GR: 100 %). Mitarbeiter aus beiden Ländern erwarten durch die Teilnahme eine erhöhte Handlungskompetenz. In Griechenland erhofft man sich einen besseren Umgang mit Menschen mit Demenz; in Deutschland werden konkrete praktische Ratschläge erwartet.

**Diskussion:**

Die Teilnahmebereitschaft an einer Schulung zu Demenz ist in beiden Ländern groß. Dabei sollte nicht nur theoretisches Wissen, sondern auch die praktische Umsetzung vermittelt werden.

## Introduction

The age of the global population is increasing, leading to an additional increase of multimorbid and frail patients who require care in general hospitals [18]. A subgroup of frail and vulnerable patients in hospitals are people with dementia (PwD) who are more likely to be admitted to general hospitals than people of similar age without dementia, particularly for fall-related accidents, chronic disease complications, urinary tract and respiratory infections [17]. Furthermore, the hospital stay is longer [15] and the outcomes are predominantly negative [8]. Specifically, it is estimated that 12.9–63.0% of people admitted to general hospitals have dementia in addition to the primary diagnosis [16]. A German study showed that at least 40% of hospital inpatients over 65 years of age have cognitive impairment [3]. A very similar proportion was revealed by a Greek study, in which cognitively impaired patients were greatly underestimated during admission to hospitals and 30.5% of older inpatients (>65 years) were diagnosed with cognitive decline [9]. The PwD usually experience poor quality of care in hospitals [21] and staff members are often insufficiently educated to meet the special needs of these patients [15]. The lack of adequate knowledge on dementia has been characterized as one of the main factors that affects care quality in hospital settings [19]. Due to this the number of dementia training programs in hospital settings is steadily increasing also internationally [19, 21, 22]. A number of factors regarding an effective dementia education for hospital staff have already been identified in the literature. Effective training programs appeared to be longer than 8 h duration and were classroom-based. Moreover, training content must be relevant to participants’ work, while practical tools and care strategies must be directly applicable in the workplace [21].

In order to develop a successful training program and enable the effective transfer of theoretical education to practice, an educational needs analysis should be carried out with respect to the target group [13, 19, 24]. Specifically, where trainees are involved and given choices about training processes, the training should influence their motivation to learn. Thus, an increase in trainees’ attention during training might possibly lead to the successful clinical implementation of what they learned [14]. Not much is known about the preferences and expectations regarding future dementia training programs for Greek and German staff members working in general hospitals. Although a number of dementia training programs have already been conducted in Germany, hardly any data were published in scientific journals. To our knowledge, there have been no dementia training courses conducted in the hospital settings in Greece.

The aim of this survey was to explore the current status of nursing staff, head nurses’ and physicians’ training needs relevant to the topic of dementia in general hospital settings in Germany and Greece. Specifically, the study attempted to explore interests and expectations of nursing staff, head nurses and physicians regarding their participation in dementia training programs. This survey is part of a larger research project which supports cross-cultural research, aiming to improve the quality of hospital care for cognitively impaired patients in the abovementioned countries. In a further step, the results of this survey will be used to develop a dementia training program individually for each of the countries.

## Material and methods

### Study design

This was a descriptive survey based on a questionnaire. It was conducted from May 2017 to May 2018 in Germany and Greece.

### Settings and participants

The five hospitals located in an urban area in the south of Germany, in which the future dementia training was planned to take place, were invited to participate in this survey and three of them agreed. Participation invitation letters to hospital directors and decision-makers were sent by two authors (J.S and B.T.) via e‑mail. The two general public hospitals from an urban area in the north of Greece which agreed to participate in a future dementia training program were selected to participated in this survey. Oral invitations were given to hospital directors by one Greek author (M.G.). Finally, 2 general hospitals (1 with <250 and 1 with <500 beds) and 1 university hospital (>1800 beds) in Germany and 1 general hospital (500 beds) and 1 university hospital (690 beds) in Greece accepted to participate in the current survey.

After obtaining the official approval from hospital directors and the hospital works councils to conduct a survey both in Greece and Germany, the authors invited directors of eligible wards to participate in the survey via e‑mail or orally. Wards were selected where the treatment of many patients with secondary dementia could be suspected. According to eligibility criteria, geriatric, psychiatric and pediatric wards were excluded due to the fact that nursing and medical staff in these areas may either have special knowledge about dementia and are more aware of this topic or the topic of dementia is irrelevant to their duties. Taking the eligibility criteria into consideration, 20 wards were finally randomly recruited (cardiology, endocrinology/nephrology, gastroenterology, general surgery, general visceral surgery, hematology, internal medicine, neurosurgery, ophthalmology, orthopedics/trauma surgery, otorhinolaryngology, and pneumology). The ward sizes of the included German wards ranged between 12 and 40 beds, wards in Greece varied from 12 to 56 beds.

Nursing staff, head nurses and physicians were selected as participants because these occupational groups spend most of their time caring for PwD during hospitalization. Among these groups, no exclusion criteria of recruitment were applied. By agreement with a decision-maker of each ward, the wards were visited by researchers (J.S. and M.G.) at a randomly announced point in time. The recruitment was a random selection among those who were on duty at that time and expressed their willingness to participate. At least one person per ward from each occupational group was interviewed, whereby one German head nurse was responsible for two participating wards. A total of 61 hospital staff members participated in this survey (Germany: *n* = 25; Greece: *n* = 36).

### Questionnaire development

Because of the lack of an adequate questionnaire a tool comprising all important information for designing a future dementia training program for the hospital setting was developed. Following an intensive literature review and discussions with Greek researchers both Greek and German questionnaires were developed in parallel by an author with high proficiency in both languages (B.T.). The initial questionnaire consisted of five thematic parts: wards/hospital characteristics, participant characteristics, current care of PwD, hospital protocol regarding dementia and expectations about a dementia training program. This version was then tested in a pilot study in Greek hospitals (*n* = 24, unpublished data). The initial tool was adapted by two researchers (J.S. and M.G.) with respect to further literature on existing training programs, the systematic review (submitted for publication) and intensive discussion with all authors and other researchers with high competence in both languages in order to eliminate existing semantic divergences between the two versions. Researchers added one more thematic part about the desired characteristics of the future dementia training program (type, design, delivery). While most questions had already been tested in the Greek language during the pilot study, the German version was piloted with two nurses and one physician to ensure that there was no ambiguity in wording. The final version of the questionnaire was found to be user-friendly with no problems experienced when completing it and consisted of 54 items divided into 6 thematic groups (6 open questions and 48 closed-ended questions).

This survey analyzed items relevant to general information about the hospitals and participants, the educational background of participants, previous dementia training, preferred characteristics of a future dementia training program (type, design, delivery) and participants’ expectations on a dementia training program. The description of the analyzed items can be seen in the supplement (Supplementary material Table 1).

**Table 1:** Sample characteristics (country comparison)

	Germany *n* (%)	Greece *n* (%)
*Profession*		
Head nurse	7 (28.0)	12 (33.3)
Registered nurse	9 (36.0)	12 (33.3)
Assistant nurse	1 (4.0)	–
Senior physician	1 (4.0)	–
Assistant/junior physician	7 (28.0)	12 (33.3)
*Age (years)*		
15–25	1 (4.0)	1 (2.8)
26–35	12 (48.0)	12 (33.3)
36–45	8 (32.0)	6 (16.7)
46–55	1 (4.0)	14 (38.9)
56–65	3 (12.0)	3 (8.3)
*Years of school education*		
9 years	1 (4.0)	–
10 years	7 (28.0)	–
12 years	–	36 (100)
13 years	17 (68.0)	–
*Professional relevant further education apart from academic studies and vocational education*		
Further qualification	10 (40.0)	–
Masters degree—care	1 (4.0)	6 (16.7)
PhD or postdoctoral qualification	6 (24.0)	1 (2.8)
*Previous participation in trainings on dementia*		
Yes	6 (24.0)	2 (5.5)

25 of the German participants and 36 of the Greek participants answered these questions

### Study procedure

The survey was carried out by two researchers, one from Germany (J.S.) and one from Greece (M.G.), while a third researcher (B.T.) gave crucial supervision during the overall procedure. After receiving the survey information letter, participants gave written consent by letter. The questionnaire was assessed in a quiet setting in the staff workplace. Researchers interviewed the participants and wrote down participants anwers due to practical issues and in order to prevent possible misunderstandings during the entire procedure. At the end of each interview, the questionnaire was pseudonymized to ensure participants’ data protection. Interviews lasted 15–45 min.

### Statistical analysis

The statistical analysis was carried out using SPSS V25.0 (IBM Corp, Armonk, NY, USA). Data were analyzed with descriptive statistics. To analyze and quantify open-ended questions and open-ended response options, a thematic analysis was conducted and categories were built by the researcher team. Data were compiled, disassembled through coding, reassembled and interpreted. The categories were than quantified and transferred to SPSS V25.0. The frequencies of variables were determined.

### Ethical considerations

The survey protocol was approved by (1) the Faculty of Behavioral and Cultural Studies at Heidelberg University (Germany) and (2) Research Ethic Committees from each hospital (Greece). The survey protocol was made in accordance with the ethical standards outlined in the Declaration of Helsinki.

## Results

Each descriptive analysis of the thematic parts was divided into two main sections: a comparison between the countries and a comparison between occupational groups focusing on the key findings and differences whereby the main focus was on the country comparison. Occupational groups were grouped into three categories: nursing staff (nurse assistant and registered nurses), head nurses, and physicians (senior physician, assistant physicians and junior physicians).

### Characteristics of participants

All participants were nursing staff (*n* = 22), head nurses (*n* = 19) or physicians (*n* = 20). The majority of the Greek staff members were older (46–55 years) than the majority of the German staff members (26–35 years old). Almost half of the Greek participants and 40% of the German participants had been working in hospital settings for over 15 years. School education, foundational education and further education during their working life varied greatly between the two countries due to differences in the respective educational systems. Some Greek nurses, apart from their basic academic studies had a masters degree or a second bachelors degree (16.7%) and only one physician had a PhD degree. More than half of the German nurses had a further qualification, one nurse had a masters degree and 75% of the physicians had acquired a PhD or a postdoctoral qualification (Table 1).

In the last 5 years a high percentage of Greek participants (88.9%) had attended training courses in general outside the hospital, while only 55.6% has attended such programs within the hospital, such as conferences, seminars or workshops. Only two nurses (5.5%) had participated in a dementia education program before. The topics of these programs were: “oral hygiene in dementia” and a “multidisciplinary seminar about dementia” organized by a psychiatric department. All of the German participants had attended training courses in general within the hospital and 72% had attended such programs outside the hospital in the past 5 years. Of the attendees 6 (24%) had participated in a dementia training program (4 nurses and 2 physicians), and 1 of these nurses had become a dementia expert. The topics of the attended dementia training courses were: “dementia in general”, “communication”, “nutrition” and “medication”.

Comparing the occupational groups, the majority of physicians were younger (26–35 years old) than head nurses (46–55 years old). Nursing staff (54.5%) and head nurses (84.2%) had much more work experience than physicians as most of them had been working in hospital settings for over 15 years. The majority of physicians (65%) had worked between 1 and 5 years in the same hospital. Over 70% of each occupational group had attended training courses in general within the hospital, while more head nurses (89.5%) and physicians (95%), and less nursing staff (63.6%) had attended training courses in general outside the hospital. In the past three nursing staff and head nurses and two physicians had participated in a dementia training program (Supplementary material Table 2).

**Table 2:** Reasons for and forms of obtaining information (country comparison)

	Germany *n* (%)	Greece *n* (%)
*Obtaining information about the topic dementia independently*^*a*^		
Yes	18 (72.0)	19 (57.6)
*Reasons for obtaining information*^*b*^		
Personal interest	6 (33.3)	12 (63.2)
Employer’s specification	–	–
Current issue discussed at the workplace	10 (55.5)	5 (26.3)
PwD in the family	6 (33.3)	3 (15.8)
Other	–	2 (10.5)
*Forms of gathering information*^*c*^		
Vocational education	8 (44.4)	5 (26.3)
Seminars/workshops	7 (38.8)	5 (26.3)
Staff meetings, knowledge exchange with colleagues	9 (50.0)	2 (10.5)
Expert discussion	1 (5.6)	–
E‑learning/television	6 (33.3)	4 (21.1)
Books/journals	6 (33.3)	8 (42.1)

^a^ 25 of the German participants and 33 of the Greek participants answered this question

^b^ 18 of the German participants and 19 of the Greek participants answered this question. Multiple answers were possible

^c^ 18 of the German participants and 19 of the Greek participants answered this question. Multiple answers were possible

### Interest in the topic of dementia

The majority of the German staff members mentioned they had independently gathered information about dementia in the past (72%), while the Greek staff members were less self-informed about this topic (57.6%). Answers such as why and how they had gathered information about dementia were from a prespecified list of choices with an open-ended response option. Multiple answers were possible (Table 2). Compared to the Greek participants, a larger number of German participants used various knowledge sources to gather information about dementia and care for PwD: exchanging ideas with colleagues, seminars/workshops, e‑learning/television and books/journals were named by one third to one half of the sample, aside from the knowledge acquired during vocational education. In contrast, in the Greek sample only books or journals were mentioned by more than one third of participants. For the Germans, the reasons for gathering information were mostly current discussions at the workplace while personal interest was a predominant reason for the Greek staff members (Table 2).

The occupational analysis demonstrated that nursing staff and head nurses had independently gathered information about dementia in the past more often than physicians. Personal interest was the main reason to inform oneself about dementia for all occupational groups, whereas current discussions at the workplace were a reason for head nurses and physicians. Another important reason for head nurses to gather information about dementia was having a relative with dementia. All occupational groups used various knowledge sources to gather information about dementia and care for PwD, whereas nursing staff mostly received information during their vocational education and head nurses were using books/journals the most (Supplementary material Table 3).

### Staff preferences and expectations regarding dementia trainings

With the exception of the nursing assistant, all staff members preferred to be educated in future dementia training programs (96% for the Germans and 100% for the Greeks). Consequently, the following results sections refer to registered nurses, head nurses and physicians. Answers regarding the design of the future dementia training program were from a prespecified list of choices with an open-ended response option. Multiple answers were possible.

#### Training design

Staff members from both countries gave priority to be further educated in the form of seminars and the Germans also expressed their preference for workshops. The Greek staff members preferred to be regularly trained, whereas German staff members preferred to be trained at a longer time interval of 6 months. While German participants did not have any time preference, Greek participants preferred to be trained during their regular shift work or in full day sessions. Attendees from both countries preferred short sessions from 30 min to 45 min, while the Greek participants also mentioned a preference of 45–60 min (Tables 3 and 4).

**Table 3:** Design of a future dementia training part 1 (country comparison)^a^

	Germany *n* (%)	Greece *n* (%)
*Type of education*		
Seminars	18 (75.0)	29 (82.9)
Workshops	14 (58.3)	6 (17.2)
Staff meetings	5 (20.8)	4 (11.4)
E‑learning	2 (8.3)	2 (5.7)
Books/journals	6 (25.0)	1 (2.9)
*Frequency of training sessions*		
Once	7 (29.2)	10 (28.6)
Every 6 months	16 (66.7)	8 (22.9)
Every 3 months	2 (8.3)	1 (2.9)
Every month	1 (4.2)	2 (5.7)
Regularly	–	15 (42.9)
Other	1 (4.2)	–

^a^ 24 of the German participants and 35 of the Greek participants answered these questions. Multiple answers were possible

**Table 4:** Design of a future dementia training part 2 (country comparison)

	Germany *n* (%)	Greece *n* (%)
Time of education^a^		
*During the regular shift work*	12 (50.0)	22 (62.9)
In the morning	3 (12.5)	8 (22.9)
Midday handover time	2 (8.3)	–
In the afternoon	5 (20.8)	1 (2.9)
All day workshop	3 (12.5)	14 (40.0)
*Outside the regular shift work*	12 (50.0)	14 (40.0)
Duration of education^b^		
*30–45 min*	9 (37.5)	14 (38.9)
*45–60 min*	3 (12.5)	14 (38.9)
*60–90 min*	6 (25.0)	3 (8.3)
*Full day*	4 (16.7)	4 (11.1)
*Other*	2 (8.3)	1 (2.8)

^a^ 24 of the German participants and 35 of the Greek participants answered these questions. Multiple answers were possible

^b^24 of the German participants and 36 of the Greek participants answered these questions. Multiple answers were possible

Comparing the occupational groups, physicians did not have time preferences but preferred short individual seminars, only once, while registered nurses and head nurses were also interested in workshops, mainly in full day sessions, every 6 months, no matter when (Supplementary material Tables 4 and 5).

#### Training topics of interest

**Fig. 1:**
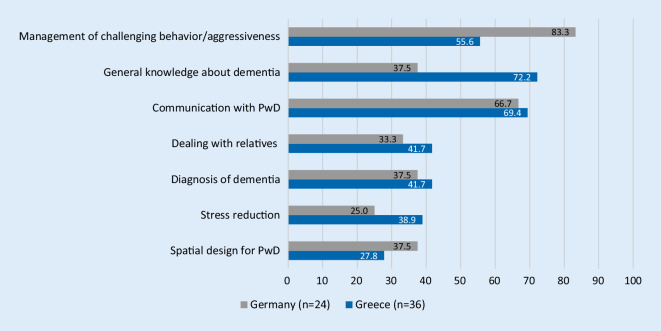
Results of the questionnaire on topics of interest in Germany and Greece in % (country comparison, multiple answers were possible)

A list of seven training topics was included in the questionnaire and provided with an open-ended response option (See Fig. 1). Multiple answers were possible. Topics of interest were predominantly similar. Participants were mostly interested in the following topics: “management of challenging behavior” and “communication with PwD”. The Greek staff members also expressed interest in learning more about “general knowledge about dementia” than the German staff members (Fig. 1).

The occupational group analysis demonstrated that all three groups (nursing staff, head nurses and physicians) were interested in learning more about “general knowledge about dementia”, “management of challenging behavior” and “communication with PwD”. Registered nurses were also interested in knowing more about the “diagnosis of dementia”. Generally head nurses and registered nurses were more interested on a variety of different topics than physicians (Supplementary material Table 5).

#### Expectations in future dementia training programs

Expectations regarding dementia training were collected by way of an open question and four main areas of expectation were identified in the respective analysis: the acquisition of clinical skills, training methodology, training topics and the trainer’s skills. Participants from both countries expected the training to enhance their clinical skills. In particular, Greek participants expect future training to teach them to handle PwD properly and thus increase their self-efficacy. Germans hoped for practical advice that can be applied immediately at the workplace. Some respondents again stated specific training topics as an expectation. Participants from both countries highlighted once again the desire to know more about dementia in general. Another area that appeared important to the respondents was the training methodology. Specifically, Greek participants expected future training to be both informative and educative, while German participants hoped for more practical oriented work-related and interactive training. The German respondents alone had expectations regarding the trainer’s skills. In particular, they expected highly experienced healthcare professionals to carry out the training program (Table 5).

**Table 5:** Identified themes and subthemes of expectations regarding a dementia training program (country comparison)^a^

Themes and subthemes	Germany		Greece	
	Number of statements	*n* (%) of participants	Number of statements	*n* (%) of participants
*Clinical skills*	*18*	*14 (60.9)*	*37*	*25 (89.3)*
Better handling of PwD	4	17.4	20	71.4
General practical advice in handling PwD	7	30.4	–	–
Increase of self-efficacy	–	–	7	25.0
Ability to advise relatives	2	8.7	6	21.4
Communication tips	3	13.0	2	7.1
Better management of challenging behavior	–	–	2	7.1
Others	2	8.7	–	–
*Training topics*	*13*	*9 (39.1)*	*12*	*7 (32.1)*
Diagnosis	–	–	4	14.3
Latest information and knowledge	3	13.0	–	–
Theoretical information about the disease	4	17.4	3	10.7
Strategies to reduce nurses’ stress	–	–	2	7.1
Others	5	21.7	4	14.3
*Training methodology*	*12*	*9 (39.1)*	*7*	*9 (25.0)*
Informative and educative training	2	8.7	6	21.4
Interactive methods	3	13.0	–	–
Practical-oriented training	3	13.0	–	–
Methods promoting reflection and increasing awareness	2	8.7	–	–
Others	2	8.7	1	3.6
*Trainer’s skills*	*3*	*3 (13.0)*	*–*	*–*
Practitioner from a clinical profession	2	8.7	–	–
Professional behavior towards participants	1	4.3	–	–

^a^ 23 of the German participants and 28 of the Greek participants answered these questions. Multiple answers were possible

Almost half of participants from each occupational group expected to enhance their clinical skills by learning the proper handling of PwD. Regarding the training topics, more physicians expected to learn more about the “diagnosis of dementia”, while nurses and head nurses needed more theoretical information about dementia. Compared to physicians, registered nurses and head nurses had more expectations for specific training methodology and trainer’s skills. Especially head nurses expected the training courses to be informative and educative (Supplementary material Table 6).

## Discussion

Exploring staff preferences about training topics, training design and general expectations is helpful to address the educational needs for future dementia training programs [24]. The responses from the participants of both countries showed interesting differences but also similarities.

Regarding the training design, the majority of the participants from both countries prefer to be trained by way of seminars at any time of the day, during or outside of their regular shift work, for 30–90 min per session. Moreover, German participants preferred short training sessions every half year, while Greek participants preferred more regularly scheduled training sessions and were open to full day sessions. Some of these findings corresponded with the characteristics of effective dementia training analyzed by Surr and Gates [21]. According to their results a duration of at least 1 day, delivered in full-day training sessions or as sessions at least for 1 h leads to positive outcomes. There might be some barriers for health staff to attend regular training sessions, for which an ongoing support via in-service experts should be provided [23]. Face-to-face seminars were more preferable than online training programs, which were hardly in demand according to the responses received, even though they were provided as response option in the utilized questionnaire. It appears that clinical staff members prefer to exchange experiences, take part in discussions and have personal contact during training programs. Some reasons could be that the use of electronic devices is still uncommon for clinic employees, or that the e‑learning method seems inappropriate for this subject area. Whereas classroom-based or face-to-face delivery in small groups are supported by the evidence [21, 23], online materials or e‑learning are discussed controversially in the literature, due to internet access problems in the workplace and difficulties with individual motivation of participants. The evidence recommends not to use e‑learning as an independent study option, e.g. in between training sessions [21].

Dealing with challenging behavior and communication were among the popular topics for both German and Greek participants. According to Dewing and Dijk [8] hospital staff members are frequently confronted by behavior such as aggression, agitation and wandering from PwD. This could possibly explain the interest in these topics. Nevertheless, the majority of the Greek participants said they preferred “general knowledge about dementia” as a topic. This finding may coincide with the fact that there has been no previous dementia training in Greek general hospitals, meaning that the staff members require more general knowledge about this topic at the very first level, whereby the German participants demanded more topic-specific knowledge; however, UK data examined by Chater and Hughes [7] showed that participants consider the imparting of the theoretical knowledge of the disease dementia to be important in order to improve provision of care.

The survey results show that training should be directly relevant to the participants’ workplace and the participants hope to acquire practical knowledge on how to handle this special patient group. Practical tools and care strategies are thus important tools for improving and dealing with PwD. According to Surr and Gates [21] the aforementioned tools and strategies are not only valuable for the learners’ reactions, they also produce positive changes and outcome in practice. The reported specific expectations regarding training methodology and trainers’ skills strengthen the existing evidence, which states that training has to be delivered by a skilled facilitator using interactive methods and practical activities, e.g. group learning, videos, scenario-based exercises and experiential learning alongside theoretical content [21, 23]. It can be emphasized that almost all of the Greek participants expect to enhance clinical skills and self-efficacy, which can probably be attributed to the fact that there has been no previous dementia training in Greek general hospitals and therefore there is an uncertainty in caring PwD. The expectations for future training programs are mostly in line with the international literature about effective training programs [21]. Already existing well-developed and evaluated training programs, such as the “Getting to Know Me” program [10, 11], the “VOICE” training course [12], or the “PCTAH” program [20] can be used as potential examples of good science and practice for Germany and Greece.

To fulfil the gold standard of good care an interprofessional team has to be involved. To provide patient-centered care dementia training programs are required to be interprofessional [4]. Therefore, expectations regarding a dementia training program should not vary too much between occupational groups. The survey results show several perspectives from hospital employees working with PwD. The interest of occupational groups in training topics does not differ greatly; however, training content and the used interactive methods and practical activities have to be directly relevant to the staff’s role and workplace [21]. Different expectations of the training design have been revealed. The authors suggest following the evidence and delivering individual sessions of not less than 1 h and additionally to provide ongoing support via in-service experts to bring the expectations of the occupational groups together.

The German participants seem to have been educated mostly in seminars or conferences conducted within the hospital, while the Greeks had attended seminars or conferences mostly outside the hospital, findings that may reveal the differences in the healthcare organizations of each country. Although both countries seem to provide a wide range of continuous training opportunities either within or outside hospitals, dementia training programs are still lacking. Dementia is a topic that must be considered more seriously in the hospital sector. According to Timmreck et al. [25], caring for PwD and generally geriatric patients will become more and more important in the future.

Dementia awareness and friendliness are two of the targets of the global dementia plan [26], but only 30 governments out of the 194 World Health Organization (WHO) member states have developed national dementia strategies [2]. In Germany a national dementia strategy is being developed at the moment [5, 6]. Greece enacted the national dementia strategy in 2014 and 2 years later, the implementation of 3 basic actions was started [1]. Educating professional caregivers in hospitals is one of the goals of national dementia strategies (Action 2, Axis 7 for the Greek national plan); however, the small number of participants who had already attended a dementia program, as well as the fact that almost all participants expressed the willingness to be trained in the future, may reflect the present lack of dementia trainings in hospital settings for both countries.

## Limitations

It should be noted that the survey was not carried out by one scientist in both countries due to language barriers. A standardized questionnaire is missing due to the exploratory approach. Furthermore, staff preferences in training program designs in general were not assessed. Recall bias should be considered in this survey. Some facts have to be highlighted that reduced the generalizability of the survey results. The data were not statistically compared but only descriptively. The sample size and the number of hospitals are small and not representative for both countries. Additionally, the included professions representing only a specific cohort of staff working in hospitals. The exclusion of psychiatric, geriatric and pediatric wards also lowers the generalizability of the results. In the future, further studies on the interests and expectations of different occupational groups should be carried out.

## Conclusion

- Individual face-to-face seminars of not less than 1 h are to be preferred on a regular basis and/or ongoing support via in-service should be provided.
- “General knowledge about dementia”, “management of challenging behavior” and “communication with PwD” should be part of the dementia training content.
- Dementia training has to be practically oriented and interactive alongside theoretical content. This supports successful implementation of the knowledge learned at hospital workplaces and promotes further development of clinical skills.
- Hospitals need to provide training opportunities with a greater emphasis on caring for PwD.

## Caption Electronic Supplementary Material

The Supplementary material includes one table about questionnaire characteristics and five tables about the occupational comparison. table 1 “Thematic areas, analysed constructs and types of items of the utilised questionnaire” table 2 “Sample characteristics (Comparison of occupations)” table 3 “Reasons for and forms of obtaining information (Comparison of occupations)” table 4 “Design of a future dementia training part 1 (Comparison of occupations)” table 5 “Design of a future dementia training part 2 (Comparison of occupations)” table 6 “Identified themes and sub-themes of expectations regarding a dementia training program (Comparison of occupations)”

## References

[1] Alzheimer Europe (2016). Greece. National dementia strategies.

[2] Alzheimer’s Disease International (2017). National dementia action plans. Examples for inspiration.

[3] Bickel H, Hendlmeier I, Heßler JB (2018). The prevalence of dementia and cognitive impairment in hospitals. Dtsch Arztebl Int.

[4] Brody AA, Galvin JE (2013). A review of interprofessional dissemination and education interventions for recognizing and managing dementia. Gerontol Geriatr Educ.

[5] Bundesministerium für Familie, Senioren, Frauen und Jugend (2016). Siebter Altenbericht. Sorge und Mitverantwortung in der Kommune – Aufbau und Sicherung zukunftsfähiger Gemeinschaften und Stellungnahme der Bundesregierung.

[6] Bundesministerium für Gesundheit (2018). Bundesgesundheitsminister Spahn und Bundesfamilienministerin Dr. Giffey starten die Entwicklung einer Nationalen Demenzstrategie. Vorstellung des Berichtes der „Allianz für Menschen mit Demenz“.

[7] Chater K, Hughes N (2013). Strategies to deliver dementia training and education in the acute hospital setting. J Res Nurs.

[8] Dewing J, Dijk S (2016). What is the current state of care for older people with dementia in general hospitals? A literature review. Dementia.

[9] Douzenis A, Michopoulos I, Gournellis R (2010). Cognitive decline and dementia in elderly medical inpatients remain underestimated and underdiagnosed in a recently established university general hospital in Greece. Arch Gerontol Geriatr.

[10] Elvish R, Burrow S, Cawley R (2014). “Getting to Know Me”: the development and evaluation of a training programme for enhancing skills in the care of people with dementia in general hospital settings. Aging Ment Health.

[11] Elvish R, Burrow S, Cawley R (2016). “Getting to Know Me”: The second phase roll-out of a staff training programme for supporting people with dementia in general hospitals. Dementia.

[12] Harwood RH, O’Brien R, Goldberg SE (2018). A staff training intervention to improve communication between people living with dementia and health-care professionals in hospital: the VOICE mixed-methods development and evaluation study. Health Services and Delivery Research.

[13] Hicks WD, Klimoski RJ (1987). Entry into training programs and its effects on training outcomes: a field experiment. Acad Manage J.

[14] Holton EF (1996). The flawed four-level evaluation model. Hum Resour Dev Q.

[15] Moyle W, Olorenshaw R, Wallis M (2008). Best practice for the management of older people with dementia in the acute care setting: a review of the literature. Int J Older People Nurs.

[16] Mukadam N, Sampson EL (2011). A systematic review of the prevalence, associations and outcomes of dementia in older general hospital inpatients. Int Psychogeriatr.

[17] Prince M, Comas-Herrera A, Knapp M, Guerchet M, Karagiannidou M (2016) World Alzheimer Report 2016. Improving healthcare for people living with dementia. Coverage, Quality and Costs now and in the Future. https://www.alz.co.uk/research/WorldAlzheimerReport2016.pdf. Accessed 10 June 2019

[18] Reynish EL, Hapca SM, de Souza N (2017). Epidemiology and outcomes of people with dementia, delirium, and unspecified cognitive impairment in the general hospital: prospective cohort study of 10,014 admissions. BMC Med.

[19] Scerri A, Innes A, Scerri C (2017). Dementia training programmes for staff working in general hospital settings—a systematic review of the literature. Aging Ment Health.

[20] Surr CA, Smith SJ, Crossland J (2016). Impact of a person-centred dementia care training programme on hospital staff attitudes, role efficacy and perceptions of caring for people with dementia: a repeated measures study. Int J Nurs Stud.

[21] Surr CA, Gates C (2017). What works in delivering dementia education or training to hospital staff? A critical synthesis of the evidence. Int J Nurs Stud.

[22] Surr CA, Gates C, Irving D (2017). Effective dementia education and training for the health and social care workforce: a systematic review of the literature. Rev Educ Res.

[23] Surr CA, Sass C, Burnley N (2018). Components of impactful dementia training for general hospital staff: a collective case study. Aging Ment Health.

[24] Tannenbaum SI, Mathieu JE, Salas E (1991). Meeting trainees’ expectations: the influence of training fulfillment on the development of commitment, self-efficacy, and motivation. J Appl Psychol.

[25] Timmreck C, Gerngras C, Klauke M et al (2017) Pflegestudie 2017. Zum Status Quo und der Zukunft von Fort- und Weiterbildungen in den Pflegeberufen. https://dpv-online.de/pdf/presse/Hochschule%20Niederrhein_Pflegestudie%202017.pdf. Accessed 10 June 2019

[26] World Health Organization (2017) Global action plan on the public health response to dementia 2017–2025. https://apps.who.int/iris/bitstream/handle/10665/259615/9789241513487-eng.pdf;jsessionid=818652DDD8E31B31679F26A5C030F342?sequence=1. Accessed 10 June 2019

