# Erratum to: Expectations of nursing personnel and physicians on dementia training: A descriptive survey in general hospitals in Germany and Greece

**DOI:** 10.1007/s00391-021-01889-5

**Published:** 2021-04-21

**Authors:** Julia Schneider, Mara Gkioka, Sotirios Papagiannopoulos, Despina Moraitou, Brigitte Metz, Magdalini Tsolaki, Andreas Kruse, Birgit Teichmann

**Affiliations:** 1 Network Aging Research, Heidelberg University, Bergheimer Straße 20, 69115 Heidelberg, Germany; 2 School of Medicine, Aristotle University of Thessaloniki, University Campus, 54124 Thessaloniki, Greece; 3 3rd Department of Neurology, Aristotle University of Thessaloniki, Thessaloniki, Greece; 4 Lab of Psychology, Section of Experimental & Cognitive Psychology, School of Psychology, Aristotle University of Thessaloniki, Thessaloniki, Greece; 5 Klinik für Geriatrie und Geriatrisches Zentrum Karlsruhe, Diakonissenkrankenhaus Karlsruhe-Rüppurr, ViDia Christliche Kliniken Karlsruhe, Karlsruhe, Germany; 6 1st Department of Neurology, AHEPA University Hospital, Thessaloniki, Greece; 7 Insitute of Gerontology, Heidelberg University, Heidelberg, Germany

**Erratum to:**

**Z Gerontol Geriat 2019**

10.1007/s00391-019-01625-0

Please note the correct tables of the electronic supplementary material. The original article has been corrected.

## Supplementary Information

The Supplementary material includes one table about questionnaire characteristics and five tables about the occupational comparison.

