# Supplementary material for: Expectations of nursing personnel and physicians on dementia training: A descriptive survey in general hospitals in Germany and Greece
Source: Z Gerontol Geriatr. 2019 Oct 15;52(Suppl 4):249–57. doi: 10.1007/s00391-019-01625-0 (PMC6821659; doi:10.1007/s00391-019-01625-0)
Supplement: Supplementary file 1 — The Supplementary material includes one table about questionnaire characteristics and five tables about the occupational comparison. table 1 “Thematic areas, analysed constructs and types of items of the utilised questionnaire” table 2 “Sample characteristics (Comparison of occupations)” table 3 “Reasons for and forms of obtaining information (Comparison of occupations)” table 4 “Design of a future dementia training part 1 (Comparison of occupations)” table 5 “Design of a future dementia training part 2 (Comparison of occupations)” table 6 “Identified themes and sub-themes of expectations regarding a dementia training program (Comparison of occupations)” [file 391_2019_1625_MOESM1_ESM.docx]

Supplementary table 1: Thematic areas, analysed constructs and types of items of the utilised questionnaire.

| Thematic areas | Constructs | Types of items |
| --- | --- | --- |
| Interest in the topic of dementia | **Obtaining information about the topic of dementia independently** | Yes or no answer  Closed-ended |
|  | **Reasons for obtaining information** | 5 items: 4 statements and 1 open-ended response option  Multiple answers were possible |
|  | **Type of obtaining information** | 7 items: 6 statements and 1 open-ended response option  Multiple answers were possible |
| Training design | **Desire to be trained in the topic of dementia** | Yes or no answer  Closed-ended |
|  | **Type of training** | 6 items: 5 statements and 1 open-ended response option  Multiple answers were possible |
|  | **Frequency of training sessions** | 5 items: 5 statements  Multiple answers were possible |
|  | **Time period for attending a training** | 6 items: 6 statements  Multiple answers were possible |
|  | **Duration of training sessions** | 4 items: 3 statements and 1 open-ended response option  Multiple answers were possible |
| Training topics of interest | **Topics of a dementia training program** | 8 items: 7 statements and 1 open-ended response option  Multiple answers were possible |
| Expectations in future dementia training programs | **Expectations** | 2 items: 2 statements with space for explanation |

Supplementary table 2: Sample characteristics (Comparison of occupations)^1^.

|  | | Nursing staff  *n* (%) | Head nurses  *n* (%) | Physicians  *n* (%) |
| --- | --- | --- | --- | --- |
| **Age** | | | | |
|  | 15-25 | 2 (9.1) | - | - |
|  | 26-35 | 5 (22.7) | 2 (10.5) | 17 (85.0) |
|  | 36-45 | 8 (36.4) | 3 (15.8) | 3 (15.0) |
|  | 46-55 | 5 (22.7) | 10 (52.6) | - |
|  | 56-65 | 2 (9.1) | 4 (21.1) | - |
| **Years of school education** | | | | |
|  | 9 years | 1 (4.5) | - | - |
|  | 10 years | 4 (18.2) | 3 (15.8) | - |
|  | 12 years | 12 (54.5) | 12 (63.2) | 12 (60.0) |
|  | 13 years | 5 (22.7) | 4 (21.1) | 8 (40.0) |
| **Work experience in hospitals** | | | | |
|  | 1-5 years | 2 (9.1) | - | 13 (65.0) |
|  | 6-10 years | 3 (13.6) | 1 (5.3) | 5 (25.0) |
|  | 11-15 years | 5 (22.7) | 2 (10.5) | 1 (5.0) |
|  | >15 years | 12 (54.5) | 16 (84.2) | 1 (5.0) |
| **Previous participation in trainings on dementia** | | | | |
|  | Yes | 3 (13.6) | 3 (15.8) | 2 (10.0) |
| ^1^ 22 nursing staff, 19 head nurses and 20 physicians answered this question. | | | | |

Supplementary table 3: Reasons for and forms of obtaining information (Comparison of occupations).

|  | | Nursing staff  *n* (%) | Head nurses  *n* (%) | Physicians  *n* (%) |
| --- | --- | --- | --- | --- |
| **Obtaining information about dementia independently^1^** | | | | |
|  | Yes | 13 (65.0) | 14 (73.7) | 10 (52.6) |
| **Reasons for obtaining information^2^** | | | | |
|  | Personal interest | 8 (61.5) | 5 (35.7) | 5 (50.0) |
|  | Employer’s specification | - | - | - |
|  | Current issue discussed at the workplace | 3 (23.1) | 6 (42.9) | 6 (60.0) |
|  | PwD in the family | 2 (15.4) | 6 (42.9) | 1 (10.0) |
|  | Other | 1 (7.7) | - | 1 (10.0) |
| **Forms of information gathering^3^** | | | | |
|  | Foundational education | 7 (58.3) | 3 (21.4) | 3 (27.3) |
|  | Seminars/workshops | 3 (25.0) | 4 (28.6) | 5 (45.5) |
|  | Staff meetings, exchange knowledge with colleagues | 2 (16.7) | 5 (35.7) | 4 (36.4) |
|  | Expert discussion | - | 1 (7.1) | - |
|  | E-learning/television | 6 (50.0) | 3 (21.4) | 1 (9.1) |
|  | Books/journals | 3 (25.0) | 6 (42.9) | 5 (45.5) |
| ^1^ 20 nursing staff, 19 head nurses and 19 physicians answered this question.  2 13 nursing staff, 14 head nurses and 10 physicians answered this question.  3 12 nursing staff, 14 head nurses and 11 physicians answered this question. | | | | |

Supplementary table 4: Design of a future dementia training part 1 (Comparison of occupations)^1^.

|  | | Nursing staff  *n* (%) | Head nurses  *n* (%) | Physicians  *n* (%) |
| --- | --- | --- | --- | --- |
| **Type of education** | | | |  |
|  | Seminars | 18 (90.0) | 14 (73.7) | 15 (75.0) |
|  | Workshops | 8 (40.0) | 8 (42.1) | 4 (20.0) |
|  | Staff meetings | 1 (5.0) | 5 (26.3) | 3 (15.0) |
|  | E-learning | 1 (5.0) | - | 3 (15.0) |
|  | Books/journals | 3 (15.0) | 2 (10.5) | 2 (10.0) |
| **Frequency of training sessions** | | | |  |
|  | Once | 5 (25.0) | 4 (21.1) | 8 (40.0) |
|  | Every 6 months | 9 (45.0) | 8 (42.1) | 7 (35.0) |
|  | Every 3 months | 1 (5.0) | - | 2 (10.0) |
|  | Every month | 2 (10.0) | 1 (5.3) | - |
|  | Regularly | 5 (25.0) | 6 (31.6) | 4 (20.0) |
|  | Other | 1 (5.0) | - | - |
| ^1^ 20 nursing staff, 19 head nurses and 20 physicians answered this question. | | | | |

Supplementary table 5: Design of a future dementia training part 2 (Comparison of occupations).

|  | | Nursing staff  *n* (%) | Head nurses  *n* (%) | Physicians  *n* (%) |
| --- | --- | --- | --- | --- |
| **Time of education^1^** | | | |  |
|  | During the regular shift work | 10 (50.0) | 14 (73.7) | 10 (50.0) |
|  | - In the morning | 3 (15.0) | 3 (15.8) | 5 (25.0) |
|  | - Midday handover time | 1 (5.0) | 1 (5.3) | - |
|  | - In the afternoon | 1 (5.0) | 1 (5.3) | 4 (20.0) |
|  | - All-day workshop | 7 (35.0) | 8 (42.1) | 2 (10.0) |
|  | Outside the regular shift work | 11 (55.0) | 5 (26.3) | 10 (50.0) |
| **Duration of education^2^** | | | |  |
|  | 30-45’ | 6 (28.6) | 4 (21.1) | 13 (65.0) |
|  | 45-60’ | 6 (28.6) | 7 (36.8) | 4 (20.0) |
|  | 60-90’ | 5 (23.9) | 3 (15.8) | 1 (5.0) |
|  | Full day | 2 (9.5) | 4 (21.1) | 2 (10.0) |
|  | Other | 2 (9.5) | 1 (5.3) | - |
| **Training topics^3^** | | | | |
|  | General knowledge about dementia | 15 (71.4) | 10 (52.6) | 10 (50.0) |
|  | Diagnosis of dementia | 11 (52.4) | 7 (36.8) | 6 (30.0) |
|  | Management of challenging behavior | 15 (71.4) | 12 (63.2) | 13 (65.0) |
|  | Communication with PwD | 16 (76.2) | 14 (73.7) | 11 (55.0) |
|  | Spatial design | 8 (38.1) | 8 (42.1) | 3 (15.0) |
|  | Dealing with relatives | 9 (42.9) | 9 (47.4) | 5 (25.0) |
|  | Stress reduction | 8 (38.1) | 7 (36.8) | 5 (25.0) |
| ^1^ 20 nursing staff, 19 head nurses and 20 physicians answered this question.  ^2^ 21 nursing staff, 19 head nurses and 20 physicians answered this question.  ^3^ 21 nursing staff, 19 head nurses and 20 physicians answered this question. | | | | |

Supplementary table 6: Identified themes and sub-themes of expectations regarding a dementia training program (Comparison of occupations)^1^.

| **Themes and subthemes** | | **Registered Nurses** | **Head nurses** | **Physicians** |
| --- | --- | --- | --- | --- |
|  |  | Number of statements | Number of statements | Number of statements |
| **Clinical skills** | | **18** | **20** | **17** |
|  | Better handling of PwD | 8 | 8 | 8 |
|  | General practical advice in handling PwD | 3 | 1 | 3 |
|  | Increase of self-efficacy | 3 | 3 | 1 |
|  | Ability to advise relatives | 1 | 5 | 2 |
|  | Communication tips | 2 | 3 | - |
|  | Better management of challenging behavior | 1 | - | 1 |
|  | Others | - | - | 2 |
| **Training topics** | | **6** | **6** | **13** |
|  | Diagnosis | - | - | 4 |
|  | Latest information and knowledge | 1 | - | 2 |
|  | Theoretical information about the disease | 2 | 3 | 2 |
|  | Strategies to reduce nurse stress | 1 | 1 | - |
|  | Others | 2 | 2 | 5 |
| **Training methodology** | | **6** | **11** | **2** |
|  | Informative and educative training | 1 | 6 | 1 |
|  | Interactive methods | 1 | 1 | 1 |
|  | Practical oriented training | 1 | 2 | - |
|  | Methods promoting reflection and rising awareness | 2 | - | - |
|  | Others | 1 | 2 | - |
| **Trainer’s skills** | | **1** | **2** | **-** |
|  | Practitioner from a clinical profession | 1 | 1 | - |
|  | Professional behavior towards the participants | - | 1 | - |
| ^1^ 17 nursing staff, 17 head nurses and 17 physicians answered this question. Multiple answers were possible. | | | | |
